# Exogenous tryptophan application improves cadmium tolerance and inhibits cadmium upward transport in broccoli (*Brassica oleracea* var. *italica*)

**DOI:** 10.3389/fpls.2022.969675

**Published:** 2022-08-11

**Authors:** Jia Jiang, Ze Wang, Xiangzhou Kong, Yajun Chen, Jing Li

**Affiliations:** ^1^College of Animal Science and Technology, Northeast Agricultural University, Harbin, China; ^2^College of Life Sciences, Northeast Agricultural University, Harbin, China; ^3^College of Horticulture, Northeast Agricultural University, Harbin, China

**Keywords:** broccoli, cadmium, tryptophan, IAA, glucosinolate

## Abstract

Cadmium (Cd) pollution not only reduces crop yields, but also threatens human health and food safety. It is of great significance for agricultural production to improve plant Cd resistance and reduce Cd accumulation. In Arabidopsis, tryptophan (Trp) has been found to play a role in Cd resistance. However, studies on the role of exogenous Trp on Cd tolerance in crops are limited. Here, we report that exogenous Trp application can effectively alleviate biomass decline induced by Cd stress and inhibit Cd transport from roots to shoots in *Brassica oleracea* var. *italica* (broccoli). Compared to Cd stress alone, the fresh weight of shoots and roots of *B. oleracea* seedlings treated with Cd and Trp increased by 25 and 120%, respectively, and the Cd content in shoots decreased by 51.6%. In combination with physiological indices and transcriptome analysis, we preliminarily explored the mechanism of Trp alleviating Cd stress and affecting Cd transport. Trp inhibited Cd-induced indole-3-acetic acid (IAA) conjugation, thereby providing enough free IAA to sustain growth under Cd stress; Trp inhibited the indolic glucosinolate (IGS) biosynthesis induced by Cd. Considering that the synthesis of IGS consumes glutathione (GSH) as a sulfur donor, the inhibition of Trp in IGS synthesis may be conducive to maintaining a high GSH content to be against Cd stress. Consistent with this, we found that GSH content under Cd stress with Trp application was higher than that of Cd alone. In addition to alleviating the damage caused by Cd, Trp can also inhibit the upward transport of Cd from roots to shoots, possibly by repressing the expression of *HMA4*, which encodes a transporter responsible for the xylem loading and Cd upward transport.

## Introduction

Cadmium (Cd) is one of the most toxic and widespread heavy metal pollutants, affecting soil mainly through releases from agrochemicals, sewage sludge, and industrial wastes. Cd toxicity can cause various morphological, physiological, and biochemical changes in plants. Excess Cd significantly inhibits seed germination, diminishes root and shoot growth (Huybrechts and Cuypers, 2019), decreases chlorophyll contents, and disturbs photosynthetic machinery (Paunov et al., 2018). Cd stress promotes excessive production of reactive oxygen species (ROS), including superoxide radicals (O_2_^•−^), hydrogen peroxide (H_2_O_2_), and hydroxyl radicals (OH^•^) (Sandalio et al., 2001), which lead to cell damage due to lipid peroxidation. In response to this oxidative stress, plants may enhance the activity of some antioxidant enzymes (Ekmekçi et al., 2008) such as catalase (CAT), superoxide dismutase (SOD) and peroxidase (POD), and some non-enzymatic enzymes produce antioxidants such as ascorbate (AsA) and glutathione (GSH; Saud et al., 2014, 2016; Fahad et al., 2016; Amanullah et al., 2017). GSH was considered a key player in defense against metal-induced oxidative stress (Jozefczak et al., 2012). Under Cd stress, the reduced form of GSH is converted to the oxidized form of GSSG, which activates the AsA-GSH cycle, scavenges ROS, and maintains intracellular homeostasis. GSH/GSSG serves a key indicator of the redox environment and is involved in the Cd-stress-induced defense pathway (Romero-Puertas et al., 2007).

In addition to enhancing the antioxidant system, plants have evolved protective mechanisms of exclusion to alleviate Cd toxicity, including chelation, subcellular compartmentalization in vacuoles, transportation to tolerant organs or tissues, or efflux from the plant body. Therefore, the transporters involved in Cd absorption and transportation are important for Cd defense. Although Cd is not an essential element, it can be absorbed and transported by the low-affinity divalent cation transporters, including the Ca^2+^, Fe^2+^, Zn^2+^, Mn^2+^, and Cu^2+^ transporters (Clemens, 2006). Many types of transporters that regulate Cd uptake and transport have been reported, such as NRAMP family (Takahashi et al., 2011), P-type ATPase (Morel et al., 2009), ABC transporter (Gaillard et al., 2008), CAX family (Hirschi et al., 2000), ZIP family (Lasat et al., 2000), and LCT transporters (Antosiewicz and Hennig, 2004). In Arabidopsis, the ZIP transporter IRT1 and the NRAMP transporter NRAMP2 are considered to be the main entry for Cd to enter plants (Fan et al., 2014; Ismael et al., 2019). The xyloglucan endotransglucosylase/hydrolases (XTH) is involved in Cd transportation through cell wall (Ismael et al., 2019). In cells, Cd can be transported into the vacuoles by cation exchanger CAX2, CAX4, HMA3, and MRP3 (Korenkov et al., 2007). The plasma membrane heavy metal ATPase (HMA) transporters HMA4 and HMA2 contribute to Cd xylem loading and translocating from root to shoot (Verret et al., 2004; Wong and Cobbett, 2009). The responses of these transporters to Cd stress determine the absorption and translocation of this toxic ion.

Tryptophan (Trp) is an essential amino acid necessary for protein synthesis. In both animals and plants, Trp is a precursor of melatonin (N-acetyl-5-methoxytryptamine). Melatonin, a well-known multifunctional molecule in animals, has recently been found to be ubiquitously present in plants and play versatile physiological roles in plant growth, development, and protection against biotic and abiotic stresses, including Cd stress (Arnao and Hernández-Ruiz, 2019). Melatonin has been proved to protect plants from Cd toxicity by enhancing ROS scavenging and manipulating the absorption and accumulation of Cd (Gu et al., 2021). In plants, Trp is a precursor of indole-3-acetic acid (IAA), an important phytohormone. In cruciferous plants, it can also be metabolized into secondary metabolites, indolic glucosinolates (IGS), which are defense compounds against biotic stress (Halkier and Gershenzon, 2006; Ahuja et al., 2012).

Many studies have shown that Trp can promote plants’ growth and development and improve tolerance to various environmental stresses such as drought and salinity (Zemanová et al., 2014; Farooq et al., 2015). Hsiao et al. reported that the Trp synthase gene *TSB1* was induced by Cd stress and overexpression of *AtTSB1* resulted in increased Trp and improved Cd tolerance in Arabidopsis, suggesting that Trp plays a role in Cd resistance (Hsiao et al., 2007). However, studies on the role of exogenous Trp on Cd tolerance in crops are limited.

Cadmium exposure of the general population is mainly due to the intake of cereals, vegetables, and other plant-derived food (Clemens and Ma, 2016). Cruciferae plants including *B. oleracea* normally have strong ability to take up and accumulate heavy metals (Rizwan et al., 2018). The application of non-treated drain water contaminated with Cd containing industrial wastewater for irrigation of vegetable crops has enhanced Cd pollution in agricultural lands (Ahmad et al., 2021; Sardar et al., 2022). Due to the increasing Cd content in farmland soil, these vegetables face the risk of Cd pollution. Therefore, it is of great concern to reduce Cd accumulation in crops. In this study, we reported that exogenous Trp application improved Cd tolerance and inhibited Cd transport from roots to shoots in *B. oleracea*. We further performed a transcriptome analysis and revealed the possible mechanism of the beneficial effect of Trp. Our study provides a reference for better utilization of Trp to promote crop yield and resist Cd stress.

## Materials and methods

### Plant materials, Cd treatment, and Trp application

Seeds of *B. oleracea* cultivar “Youxiu” were germinated in pots containing 200 mg commercial soil Pindstrup Substrate. Pindstrup substrate containing 30% Forest Gold substrate and 70% peat was obtained from Pindstrup Horticulture Co., Ltd. China. Five seeds were sown in each pot and grown in a growth chamber under controlled conditions (25°C, relative humidity of 60–70% and 16 h photoperiod/day at an intensity of 150 μmol photons m^−2^ s^−1^) for 7 days. The plants were watered every 3 days. Since commercial soil Pindstrup Substrate is rich in nutrients, no fertilizer was applied.

*Brassica oleracea* is cultivated in many areas of China, and the soil pollution of Cd in these areas varies. In non-polluted or slightly polluted farmlands, the content of Cd in soil ranges from 0.03 to 4.7 mg/kg (Guo et al., 2021). In some polluted farmlands, Cd content reaches 22.1 mg/kg (Zhan et al., 2019). In this study, the commercial soil Pindstrup Substrate is used. The density of Pindstrup Substrate is 0.25 kg/l, which is about 1/6 of that of the field soil. Concentrations of 0, 10, 20, and 30 mg/kg Cd were used in this study (approximately equivalent to 0, 1, 3, and 5 mg/kg Cd in field soil). The concentration of Trp application is 15 mg/kg. Pre-experiments showed that 15 mg/kg was the minimum concentration that can promote plant growth, indicating that this concentration of Trp has an effective impact on plants.

Our experimental setup consisted of eight treatments. Seedlings were exposed to soils containing 0, 10, 20, and 30 mg/kg Cd with or without 15 mg/kg Trp application. The pots not supplemented with Cd and Trp were regarded as control. Tryptophan and cadmium chloride were prepared into water solutions and applied from bottom of the pots, allowing plants to absorb solution upward (Wang et al., 2019). After 7 days of treatment, the shoots (aboveground parts) and roots were harvested, respectively, for the determination of fresh weight and Cd ion content. The shoots of seedlings were used for the determination of other physiological indicators.

### Determination of chlorophyll content

Chlorophyll content in shoots was examined, as previously described by Arnon (1949).

### Determination of ROS and lipid peroxidation

Hydrogen peroxide (H_2_O_2_) and superoxide radical (O_2_^•−^) were localized histochemically by staining leaves with 3-diaminobenzidine (DAB) (to detection of H_2_O_2_) and nitroblue-tetrazolium (NBT) (to detection of O_2_^•−^), respectively, as previously described (Kumar et al., 2014). The determination of MDA was performed using the thiobarbituric acid (TBA) method, as previously described (Zhang and Huang, 2013).

### Enzyme extraction and assays

Shoots of seedlings were harvested to perform enzyme extraction and assays. The enzyme activity of SOD, POD, CAT, and APX was determined, as previously described (Mao et al., 2019).

### Determination of GSH and GSSG contents

Determination of GSH and GSSG was performed, as previously described by Ellman (1959).

### RNA extraction and transcriptome sequencing

The shoots of seedlings exposed to 0 mg/kg and 30 mg/kg Cd with or without 15 mg/kg Trp application were harvested, respectively, for RNA-seq. RNA was extracted with TRIzol reagent. The cDNA was sequenced according to the Illumina NovaSeq platform (Berry Genomics Co., Ltd., China). *Brassica oleracea* var. *italic* is a variety of *Brassica oleracea*. The genome of *Brassica oleracea* var. *oleracea*, another variety of *Brassica oleracea* served as a reference for transcriptome analysis, due to the lack of a reference genome of *Brassica oleracea* var. *italic.* Differently expressed genes (DEGs) were defined as those with *p* value < 0.05 and fold change > 2. The online Kyoto Encyclopedia of Genes and Genomes (KEGG^1^) website was used for the analysis of the enriched pathways of DEGs. The ID numbers of the key genes in IAA and IGS pathways are listed in Supplementary Table 1.

### IAA and IGS extraction and detection

The content of total IAA and free IAA was detected by Nanjing Webiolotech Biotechnology Co., Ltd. IGS was extracted by methanol and the content was determined by HPLC mass spectrometry, as previously described (Pang et al., 2009).

### Quantitative real-time PCR (qPCR) analysis

Total RNA isolated for RNA sequencing was used for qPCR analysis, as previously described (Li et al., 2019). The *ACTIN2* in *B. oleracea* was used as a reference gene. All primers used are listed in Supplementary Table 2.

### Determination of Cd ion content

An inductively coupled plasma-optical emission spectrometer (7800, Agilent, Japan) was used to determine the Cd ion content in shoots and roots. The samples were digested with an acid mixture (HNO_3_:HClO_4_ = 10:1 v/v) for 8 h and 10 h, respectively, on a 150°C hot plate. Absorbance of the filtered solution was detected by using spectrometer, and the content of Cd was calculated according to the standard curve of the corresponding ions (Zhao et al., 2018).

### Statistical analysis

Analysis of variance (ANOVA) was performed with SPSS v10.0 software (SPSS, Inc., Chicago, IL, United States). Mean values were compared with the least significant difference (LSD) between different treatments (*p* < 0.05). Data were averaged from three independent biological replicates±standard deviation (SD). Figures were plotted with Origin 2022.

## Results

### Trp application alleviated Cd’s repression on growth

**Figure 1:**
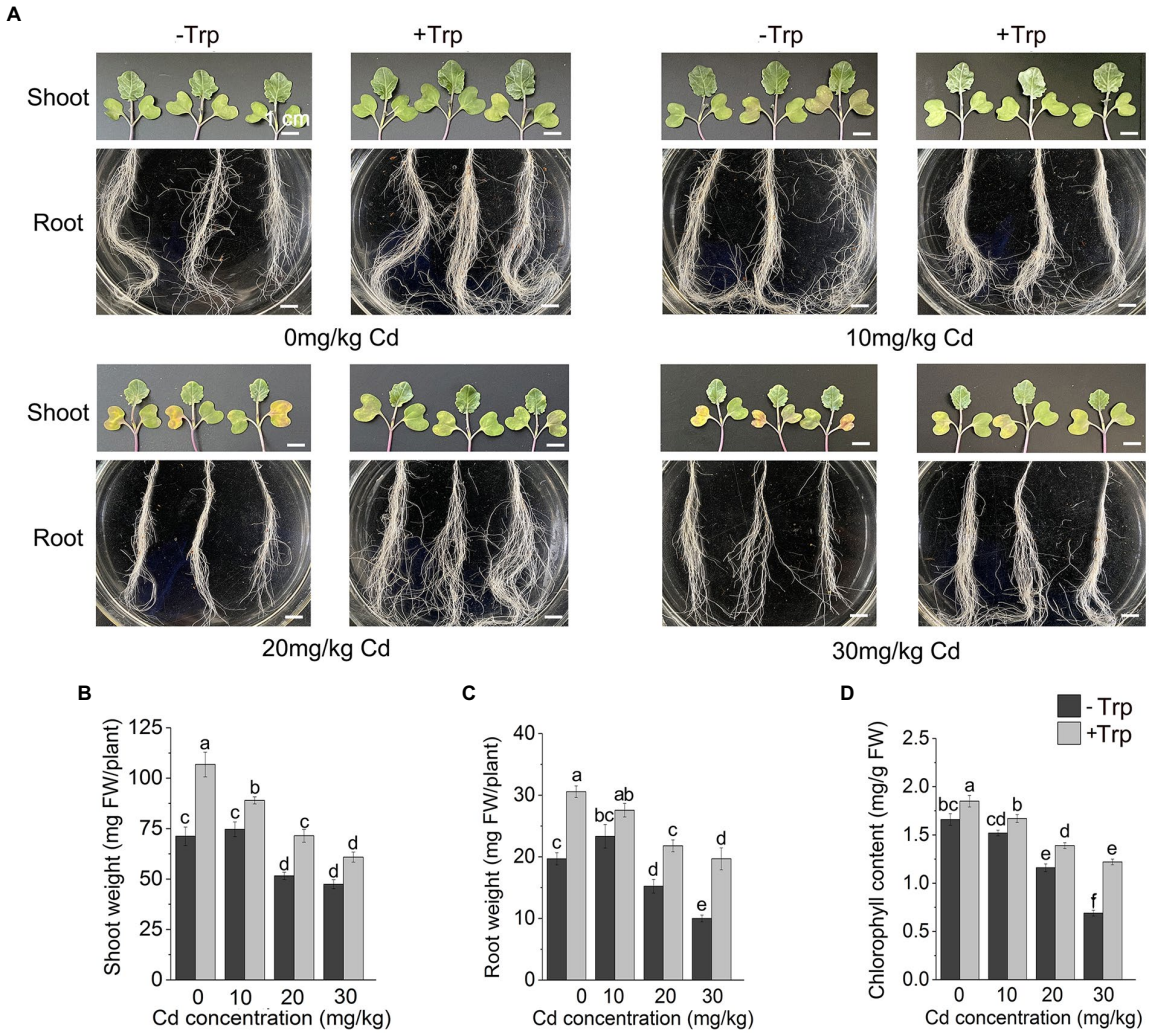
Effect of Trp on growth and development under Cd stress. **(A)** The growth of shoot and root. **(B)** Shoot weight. **(C)** Root weight. **(D)** Chlorophyll content. Data were averaged from three independent replicates ± SD. Scale bar = 1 cm. Different letters represent significant differences between different treatments (*p* ≤ 0.05). FW, fresh weight.

Under normal growth condition (0 mg/kg Cd), Trp significantly promoted the growth of *B. oleracea* seedlings (Figures 1A–C). The fresh weight of shoots and roots increased by 49 and 55.4%, respectively (Figures 1B,C). Under 10 mg/kg Cd stress, no significant fresh weight changes were observed in both shoots and roots, indicating that *B. oleracea* has strong Cd tolerance like other Cruciferae plants. Under higher Cd stress (20 mg/kg and 30 mg/kg), the growth of seedlings was significantly inhibited (Figures 1A–C), both shoot weight and root weight were reduced, and the leaves turned scorched yellow. Under 30 mg/kg Cd stress, compared to Cd stress alone, the fresh weight of shoots and roots of seedlings treated with Cd and Trp increased by 25 and 120%, respectively (Figures 1A–C). Except 10 mg/kg, 20 mg/kg, and 30 mg/kg Cd, stress significantly decreased chlorophyll content (Figure 1D). With Trp application, the chlorophyll content was higher than the unapplied controls, indicating that Trp alleviated the Cd stress-induced decrease in chlorophyll.

### Trp inhibited ROS production induced by Cd stress

**Figure 2:**
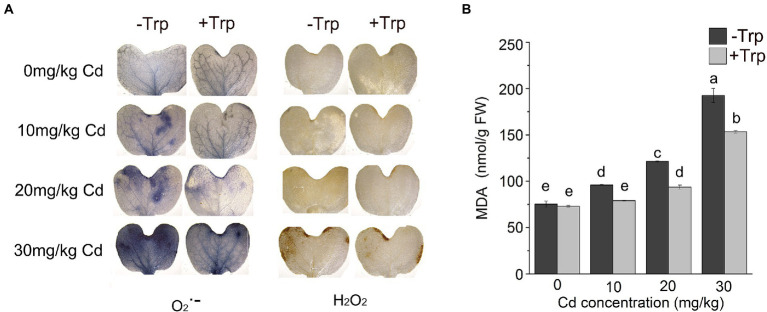
Effect of Trp on Cd induced reactive oxygen production and lipid peroxidation. **(A)** Histochemical detection of H_2_O_2_ and O^2• −^. **(B)** MDA content. Data were averaged from three independent replicates ± SD. Different letters represent significant differences between different treatments (*p* ≤ 0.05). FW, fresh weight.

As an indicator of oxidative stress, the production of H_2_O_2_ and O_2_^•–^ in *B. oleracea* seedling leaves was detected by histochemical staining. Under Cd stress, blue and brown patches representing O_2_^•–^ anions and H_2_O_2_ appeared on the leaves. As the Cd concentration increased, the patch area expanded, and the color deepened. However, Trp application decreased those patches caused by H_2_O_2_ and O_2_^•–^ noticeably (Figure 2A).

Consistent with the increased oxidative stress, MDA, the product of membrane lipid peroxidation, increased under Cd stress (Figure 2B). While Trp applied seedlings showed significantly lower MDA content compared to Cd alone (Figure 2B). These data indicated that Trp could effectively alleviate ROS-induced oxidative damage.

### Effects of Trp on antioxidant system under Cd stress

**Figure 3:**
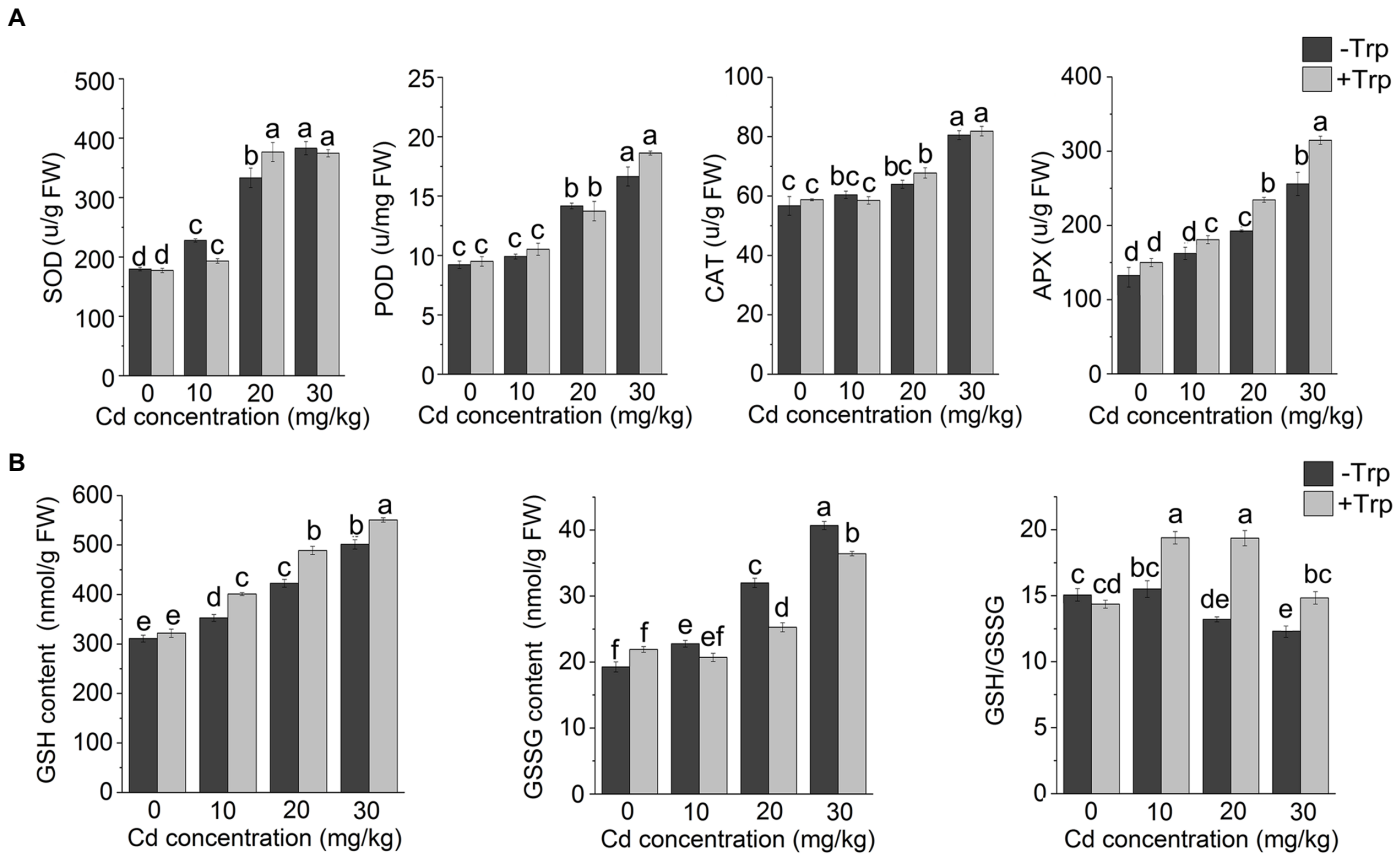
Effect of Trp on antioxidant components under Cd stress. **(A)** Activity of SOD, POD, CAT and APX. **(B)** GSH, GSSG and GSH/GSSG. Data were averaged from three independent replicates ± SD. Different letters represent significant differences between different treatments (*p* ≤ 0.05). FW, fresh weight.

As shown in Figure 3A, Cd stress increased the activity of SOD, POD, and CAT. However, application of Trp did not appear to affect the response of these antioxidant enzymes to excess Cd as the seedlings applied with Trp showed no altered enzyme activity compared to those treated with Cd alone. In contrast, the response of APX to Cd stress was significantly affected by Trp. The activity of APX was enhanced by Cd stress, and the increase in APX activity was greater in Trp-treated seedlings. GSH was considered a key player against metal-induced oxidative stress. Thus, GSH, GSSG, and GSH/GSSG were detected under Cd stress with or without Trp application. As shown in Figure 3B, both GSH and GSSG content increased under Cd stress. While in the presence of Trp, GSH increased more, and GSSG increased less than that of Cd alone. As a result, GSH/GSSG was significantly higher in Trp applied seedlings. These results indicated that the Trp application promoted Cd-induced GSH production and thus alleviated oxidative stress.

### Effect of Trp on transcriptome under Cd stress

**Figure 4:**
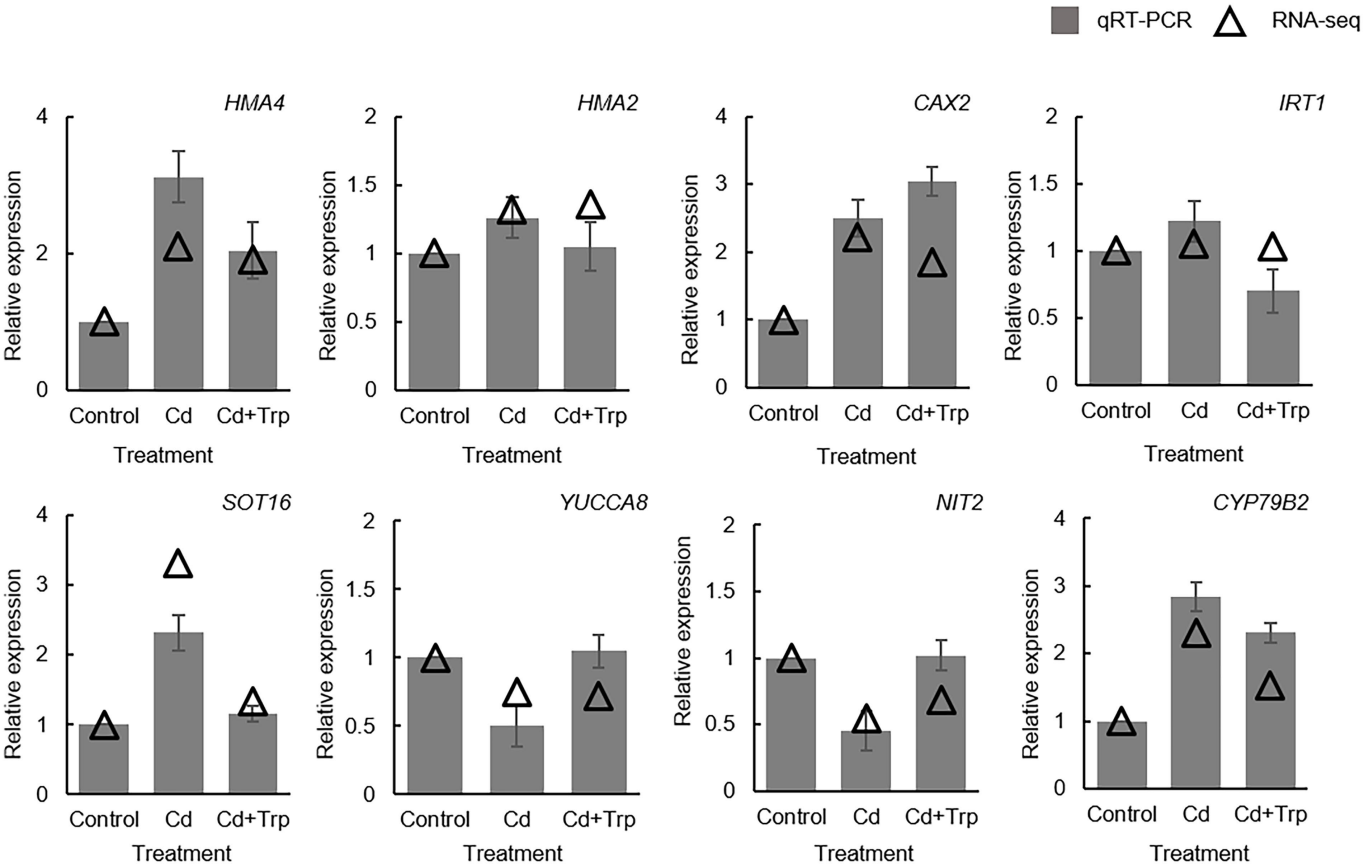
Validation of transcriptome data by qRT-PCR. Expression value of the control sample without treatment is set to 1. Relative genes expression levels in comparison with the control are shown. Data were averaged from three independent replicates ± SD.

To further elucidate the mechanism of Trp in alleviating Cd stress, we performed transcriptome sequencing analysis on *B. oleracea* seedlings exposed to 0 mg/kg and 30 mg/kg Cd with or without Trp application. Eight genes were selected for qRT-PCR detection to confirm the reliability of transcriptome sequencing. As shown in Figure 4, the expression profiles of these genes were largely consistent with those derived from sequencing, indicating that our analysis based on transcriptome data was reliable.

**Figure 5:**
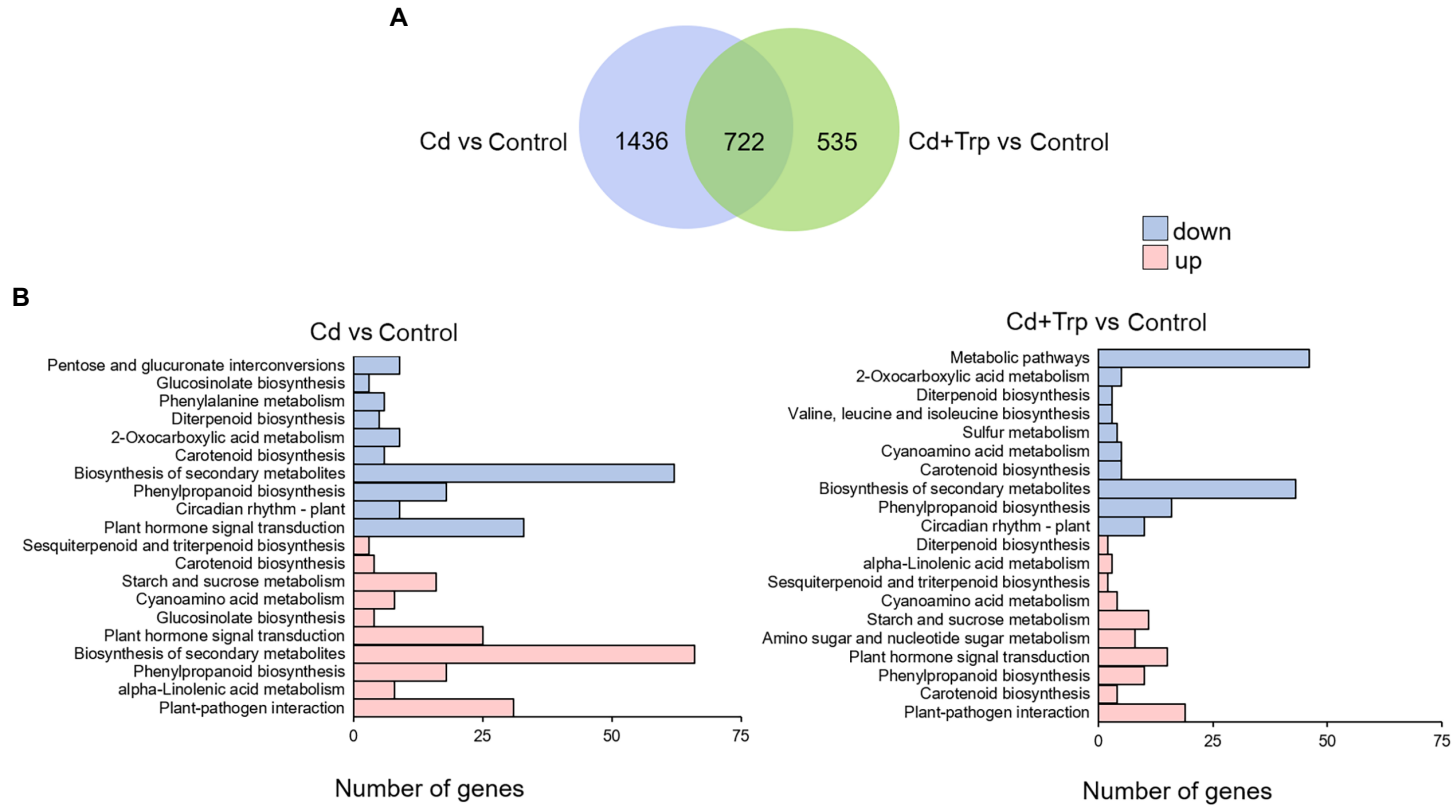
Analysis of differentially expressed genes (DEGs) and KEGG enrichment under Cd stress with or without Trp applicaiton. **(A)** Venn diagram of the distribution of DEGs responsive to Cd stress with or without Trp application. **(B)** KEGG pathway enrichment analysis of DEGs responsive to Cd stress with or without Trp application.

As shown in Figure 5A, the number of DEGs detected in seedlings treated with Cd alone was 2,158, while the number in the seedlings treated with Cd and Trp was 1,257. This result indicated that the responses of *B. oleracea* seedlings triggered by Cd with Trp application were less intense than Cd alone. Application of Trp, alleviated to some extent the stimulation of Cd. KEGG analysis showed that under Cd treatment, for both upregulated and downregulated DEGs, the most enriched pathway was “secondary metabolites biosynthesis” (Figure 5B). It is noteworthy that “glucosinolate biosynthesis” was presented in both the upper and lower DEGs, which indicated that the secondary metabolite glucosinolate is closely related to Cd defense. Interestingly, the “glucosinolate biosynthesis” pathway was not enriched under Cd treatment with Trp application. These results suggested that glucosinolate metabolism was critical for defense against Cd stress, and was strongly affected by exogenous Trp. As shown in Figure 5B, “plant hormone signal transduction” was another largely enriched pathway under Cd stress. However, with the application of Trp, the downregulated DEGs in this pathway were not enriched and the number of upregulated DEGs enriched in this pathway was smaller than that under Cd treatment alone. This indicated that hormone signal transduction was largely affected by Trp under Cd stress.

### Effects of Trp on its downstream metabolic network under Cd stress

**Figure 6:**
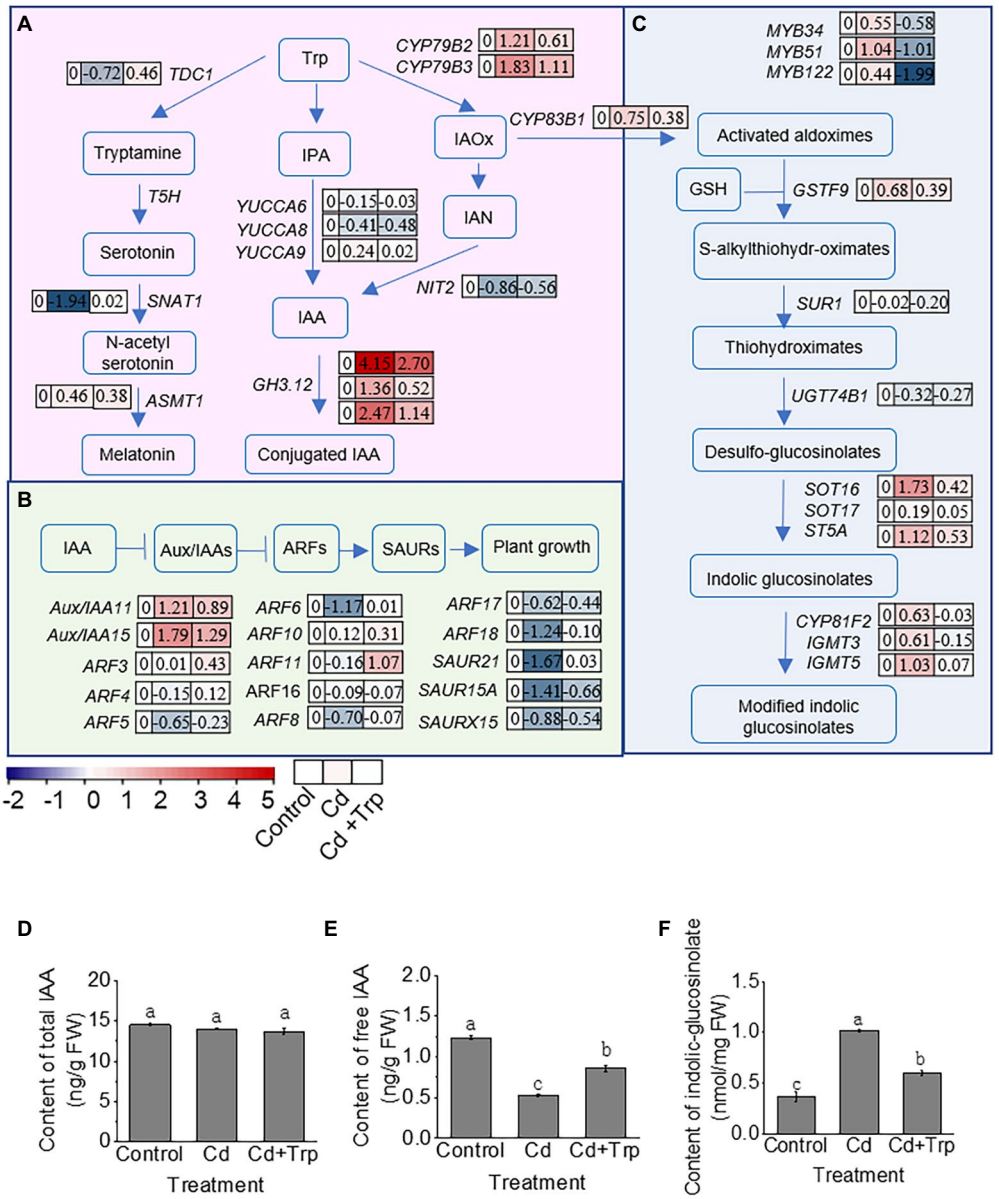
Effect of Trp on its downstream metabolic pathways under Cd stress. **(A)** Schematic diagram of metabolic pathway of IAA and melatonin. **(B)** Schematic diagram of signaling pathway of IAA. **(C)** Schematic diagram of biosynthesis of indolic glucosinolates. MYB51, MYB34, and MYB122 are transcription factors that activate the expression of genes in indolic glucosinolates biosynthesis. Heat map of the expression profiles is shown next to the name of the genes. Values indicate log_2_ of the gene transcript levels under Cd stress with or without Trp application versus that of control. **(D)** Total IAA content. **(E)** Free IAA content. **(F)** Indolic glucosinolates content. Trp, tryptophan; IPA, indole-3-pyruvic acid; IAOx, indole-3-acetaldoxime; IAN, indole-3-acetonitrile; IAA, indole-3-acetic acid; Aux/IAA, Aux/IAA family; ARF, auxin response factor; SAUR, small auxin up RNA. FW, fresh weight. Data were averaged from three independent replicates ± SD.

In plants, Trp is a precursor of hormone IAA, antistress compound melatonin, and secondary metabolites IGS (Figure 6A). To explore the effect of Trp on its downstream metabolic network, the gene expression in melatonin, IAA, and IGS metabolic pathways was analyzed based on transcriptome data.

In plants, melatonin is synthesized from Trp *via* tryptamine, serotonin, and N-acetylserotonin by four enzymes TDC1, T5H, SNAT1, and ASMT1. As shown in Figure 6A, in this pathway, the expression of *TDC1* and *SNAT1* was significantly inhibited by Cd, while the expression of the two genes hardly changed when Trp was applied with Cd. This indicated that the biosynthesis of melatonin and the protective effect mediated by melatonin was restrained under Cd stress, while Trp application can alleviate this restriction.

Trp can be metabolized to IAA *via* the indole-3-pyruvate (IPA) pathway and the indole-3-acetaldehyde oxime (IAOx) pathway. The key enzymes in IPA pathway are YUCCAs, and in the IAOx pathway are NITs (Figure 6A). Based on transcriptome data, we analyzed the expression profiles of key enzyme genes involved in IAA biosynthesis, catabolism, and signal transduction. As shown in Figure 6A, the expression level of three *YUCCA* genes and one *NIT* gene was not altered or slightly decreased under Cd stress, and showed no significant difference under Cd treatment with Trp application compared with Cd alone.

In addition to biosynthesis, IAA homeostasis can also be regulated by amino acid conjugation catalyzed by amino synthetase, GH3. Unlike free IAA, the conjugated IAA is unable to activate IAA signaling and may be further degraded. As shown in Figure 6A, the expression of three *GH3* genes was significantly increased under Cd stress, while Trp application significantly alleviated this increase. This indicated that Trp application did not significantly affect IAA biosynthesis, but inhibited the conjugation of IAA induced by Cd stress and thus ensured adequate free IAA to activate IAA signaling. Consistently, the expression profile of the genes in IAA signaling pathway indicated that IAA signaling was significantly inhibited under Cd stress, while remained unaltered or only slightly inhibited under Cd stress with Trp application (Figure 6B).

In addition to serve as a precursor of IAA, Trp can also be catalyzed to the secondary metabolite IGS. First, Trp is converted to IAOx by CYP79B2 and CYP79B3, IAOx is then catalyzed to IGS or modified IGS by a series of oxidase, lyase, and transferase including CYP83B1, GSTF9, SUR1, UGT74B1, SOT16, SOT17, ST5A, CYP81F2, IGMT3, and IGMT5. The biosynthesis of IGS is mainly activated by three transcription factors MYB51, MYB34, and MYB122. As shown in Figure 6C, under Cd stress, the expression level of *MYB51*, *MYB34,* and *MYB122* increased, while under Cd stress with Trp application, the expression of the three *MYB* genes decreased. Consistent with this, the expression level of most biosynthetic enzyme genes was increased under Cd stress, while remained almost unchanged or slightly increased with Trp application. This indicated that Trp application inhibited Cd stress-induced IGS biosynthesis. It was noteworthy that GSH, the key chelator and antioxidant against Cd stress, participates in IGS biosynthesis as a sulfur donor (Figure 6C). Thus, inhibition of Trp on IGS synthesis may help maintain high GSH content to be against Cd stress. In consistent with this hypothesis, the GSH content under Cd stress with Trp application was higher than that of Cd alone, as shown in Figure 3B.

To verify whether gene expression can represent the metabolism of IAA and IGS based on transcriptome analysis, the contents of IAA and IGS were detected. As shown in Figures 6D,E, the total IAA content remained unchanged under Cd stress with or without Trp application. While as the free IAA content under Cd stress with Trp application was significantly higher than that of Cd alone. The IGS content increased significantly under Cd stress (Figures 6F). However, with Trp application, the IGS content was lower than that of Cd alone. This suggested that gene expression based on transcriptome analysis well reflected the changes in the IAA and IGS pathways.

### Effect of Trp on the transport and accumulation of Cd

**Figure 7:**
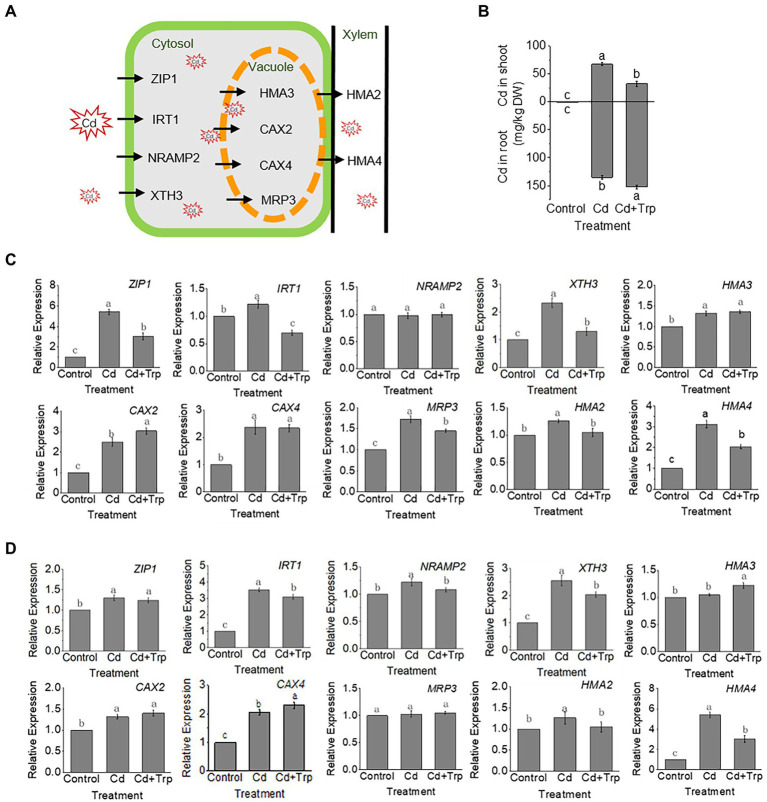
Effects of Trp on Cd accumulation and transport under Cd stress. **(A)** Schematic diagram of key proteins involved in Cd transport. **(B)** Contents of Cd in shoot and root. **(C)** Expression of transporter genes in shoot. **(D)** Expression of transporter genes in root. Expression value of the control sample without treatment is set to 1. Relative gene expression values in comparison with the control are shown. Data were averaged from three independent replicates ± SD. Different letters represent significant differences between different treatments (*p* ≤ 0.05).

According to transcriptome analysis, expression levels of most genes involved in Cd transport (Figure 7A) increased under Cd stress with or without Trp application. Compared to Cd alone, the expression level of most of these genes was lower under Cd with Trp application, indicating that the translocation of Cd was influenced by Trp. To verify this hypothesis, the Cd content in shoots and roots was measured. As shown in Figure 7B, Cd content was lower in shoots and higher in roots in the seedlings treated with Cd and Trp than those treated with Cd alone, suggesting that Trp inhibited the transport of Cd from roots to shoots. Since the transcriptome data were obtained from the shoots of the seedlings (Figure 7C) and cannot reflect the gene expression profile in root, we further detected the expression of the transporter genes in root by qRT-PCR (Figure 7D). For the proteins responsible for transporting Cd into cells (ZIP1, IRT1, NRAMP2, and XTH3), and the cation exchangers transporting Cd into vacuoles (HMA3, CAX2, CAX4, and MRP3), the expression levels of these genes showed no or little difference between Cd stress with Trp application and Cd stress alone (Figure 7D).

HMA2 and HMA4 are transporters for xylem loading and upward transport of Cd. As shown in Figure 7D, *HMA4* expression increased significantly under Cd stress with or without Trp; however, the expression level under Cd stress with Trp application was significantly lower than that of Cd alone. This suggested that Trp might inhibit the loading of Cd into the xylem and upward transport of Cd to shoots.

## Discussion

Cd is a recognized heavy metal whose accumulation in crops and possible entrance into consumer’ diets may raise serious public health concerns. Various methods such as biochar, compost, manures, and silicon have been used to reduce Cd uptake and toxicity in crops (Rizwan et al., 2012; Ali et al., 2013; Rehman et al., 2015; Baldantoni et al., 2016; Sun et al., 2016). As a precursor of phytohormone IAA, Trp is often used to improve crops’ yield and fitness. It also has been observed to have an effect on Cd toxicity (Hsiao et al., 2007; El-Sayed et al., 2018, 2019; El-Shanhorey and Ahmed, 2021). However, the role of Trp in the regulation of Cd stress at the molecular level is not clearly understood. In this study, we found that exogenous Trp application can effectively alleviate the damage caused by Cd stress and inhibit the transport of Cd from roots to shoots in *B. oleracea*. Through transcriptome analysis, we preliminarily explored the mechanism by which Trp alleviates Cd toxicity and affects Cd transport.

Cd stress induces excessive accumulation of ROS and causes oxidative damage. Our study showed that Trp application effectively inhibited the accumulation of H_2_O_2_ and O_2_^−^ and thus alleviated oxidative damage induced by Cd. Under Cd stress, the activity of APX was induced, and Trp enhanced this induction. APX is considered to be a critical peroxidase for scavenging H_2_O_2_ in plants, and plays an important role in response to multiple environmental stresses (Nounjan et al., 2012). Therefore, Trp-mediated H_2_O_2_ scavenging under Cd stress may be dependent on APX. Interestingly, Trp promoted APX activity under Cd stress, but did not affect APX activity without Cd stress. The possible explanation is that APX activity is induced by H_2_O_2_ (Volkov et al., 2006; Yao et al., 2021), and in the absence of Cd, whether Trp is applied or not, the amount of H_2_O_2_ will not increase (Figure 2).

Transcriptome analysis of *B. oleracea* seedlings exposed to Cd with or without Trp indicated that secondary metabolites (particularly glucosinolates) and plant hormone signal transduction were strongly responsive to Cd and were significantly affected by Trp (Figure 5). Since Trp is the precursor of plant hormone IAA and the precursor of secondary metabolite indolic glucosinolates (IGS), the metabolism pathway of IAA and IGS was selected for further analysis.

The dynamic balance of phytohormone IAA metabolism plays an important role in regulating plant growth and development (Ludwig-Müller et al., 2021). As a precursor of IAA, exogenous Trp normally increases the IAA level and promotes plant growth and development (Dahab and El-Aziz, 2006). Therefore, Trp is often used as a substitute for IAA to improve crop yield and promotes tolerance to various environmental stresses (Zemanová et al., 2014; Farooq et al., 2015). In this study, Trp effectively alleviated the biomass reduction of *B. oleracea* seedlings exposed to Cd. However, gene expression analysis showed that under Cd stress, exogenous Trp did not flow more to IAA biosynthesis pathway, but inhibited IAA conjugation to maintain the amount of free IAA and keep plant growth.

Most detoxification processes of plants are related to the synthesis of sulfur-containing compounds, such as GSH and phytochelatin, thus increasing the plants’ requirement for sulfur (Ernst et al., 2010). IGS is a sulfur-containing secondary metabolite, whose biosynthesis requires GSH as a sulfur donor. It has been frequently reported that the biosynthesis of IGS was responsive to Cd stress (Jakovljević et al., 2013; Durenne et al., 2017), although the physiological significance of this response is not clear. Our study found that Trp inhibited Cd-induced IGS biosynthesis. We speculate that Trp-mediated inhibition of IGS synthesis under Cd stress can reduce consumption of GSH, a key regulator against Cd stress. This may be the important mechanism by which Trp alleviates Cd toxicity.

In plants, Trp is not only the precursor of IAA and IGS, but also can be metabolized into melatonin. Melatonin, a molecule used to be considered to play important functions in animals, has recently been shown pleiotropic physiological roles in plants. It is considered to be a new plant hormone because of the recent identification of the plant melatonin receptor (Arnao and Hernández-Ruiz, 2019). A large number of studies have proved that melatonin functions as a protective agent against multiple stress conditions. Melatonin can protect plants from Cd toxicity by enhancing ROS scavenging and manipulating the absorption and accumulation of Cd (Hasan et al., 2019; Gu et al., 2021). In this study, Trp alleviated the inhibition of melatonin biosynthesis caused by Cd. Considering the mechanism by which melatonin protects plants against Cd toxicity, the effect of Trp on alleviating Cd-induced oxidative stress and inhibiting Cd accumulation is likely to be depend on melatonin.

The accumulation and distribution of Cd is related to transporters belonging to various families such as ZIP, ABC, NRAMP, and P-type ATPase (Lasat et al., 2000; Gaillard et al., 2008; Morel et al., 2009; Takahashi et al., 2011). Among these transporter genes, expression of *HMA4* in root was induced by Cd stress and Trp significantly inhibits this induction. HMA4 (Heavy Metal ATPase4) is a member of the P-type ATPases family, which are transporters that utilize the energy liberated from the exergonic ATP hydrolysis reaction in order to translocate positively charged substrates across membranes (Axelsen and Palmgren, 2001). *HMA4* encodes a Zn/Cd pump that mediates metal translocation from root to shoot (Mills et al., 2005). *HMA4* overexpression increases shoot Cd levels, while *HMA4* knock-out, in contrast, decreases shoot Cd levels (Verret et al., 2004; Liedschulte et al., 2017). HMA4 is considered as the key enzyme for root-to-shoot Cd translocation and a major genetic determinant for Cd tolerance (Verret et al., 2004; Liedschulte et al., 2017). Based on the above analysis, Trp may inhibit Cd up-transport by repressing the expression of *HAM4*.

This study proved that Trp application can effectively alleviate the loss of biomass of *B. oleracea* and inhibit the upward transport of Cd to the shoot. Tryptophan is an essential amino acid for protein synthesis in plants, animals, and microorganisms. The application of Trp in soil can not only promote the growth of plants, but also be beneficial to rhizosphere microorganisms (Lebrazi et al., 2020) and safe to animals. In addition, Trp can be produced at low cost by microbial fermentation. Thus, it is economical and safe to use Trp to alleviate Cd toxicity and prevent Cd accumulation in crops.

## Conclusion

**Figure 8:**
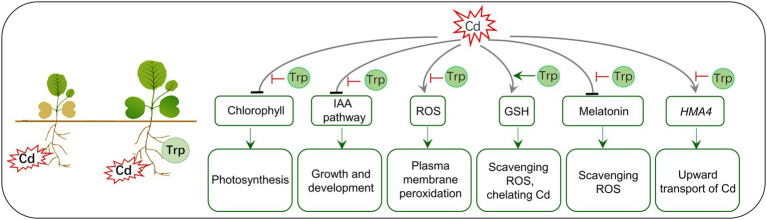
A hypothetical model for Trp application affecting toxicity and transport of Cd.

Trp is the precursor of the phytohormone IAA and some antistress molecules (melatonin and glucosinolates) and therefore is often used to improve crops’ yield and fitness. In this study, we found that exogenous Trp application can effectively alleviate the reduction of biomass caused by Cd stress and inhibit the transport of Cd from roots to shoots in *B. oleracea*. In Figure 8, a hypothesis of Trp affecting the toxicity and transport of Cd is proposed. (1) Trp inhibits Cd-induced chlorophyll degradation and auxin signaling blockage, thus alleviating growth inhibition and biomass loss. (2) Trp prevents the excessive accumulation of ROS triggered by Cd, protecting the plasma membrane from damage. (3) Under Cd stress, Trp promotes the production of GSH, which is a potent antioxidant, heavy metal chelator, and signal molecule. (4) Cd represses the biosynthesis of melatonin, a multifunctional antistress molecule, while Trp alleviates the repression. (5) Trp prevents the transport of Cd from roots to shoots by inhibiting the expression of transporter gene *HAM4*.

## Data availability statement

The original contributions presented in the study are publicly available. This data can be found here: NCBI, PRJNA863117.

## Author contributions

JJ performed the experiment and wrote the manuscript. ZW performed the experiment. XK collected and analyzed the data. YC wrote the manuscript. JL designed the experiment and wrote the manuscript. All authors contributed to the article and approved the submitted version.

## Funding

This work was financially supported by the National Natural Science Foundation of China (31570298).

## Conflict of interest

The authors declare that the research was conducted in the absence of any commercial or financial relationships that could be construed as a potential conflict of interest.

## Publisher’s note

All claims expressed in this article are solely those of the authors and do not necessarily represent those of their affiliated organizations, or those of the publisher, the editors and the reviewers. Any product that may be evaluated in this article, or claim that may be made by its manufacturer, is not guaranteed or endorsed by the publisher.

## Supplementary material

The Supplementary material for this article can be found online at: https://www.frontiersin.org/articles/10.3389/fpls.2022.969675/full#supplementary-material

## Footnotes

^1^ https://www.genome.jp/kegg/

## References

[ref1] Ahmad A. Yasin N. A. Khan W. U. Akram W. Wang R. Shah A. A.. (2021). Silicon assisted ameliorative effects of iron nanoparticles against cadmium stress: attaining new equilibrium among physiochemical parameters, antioxidative machinery, and osmoregulators of *Phaseolus lunatus*. Plant Physiol. Biochem. 166, 874–886. doi: 10.1016/j.plaphy.2021.06.016, PMID: 34237605

[ref2] Ahuja I. Kissen R. Bones A. M. (2012). Phytoalexins in defense against pathogens. Trends Plant Sci. 17, 73–90. doi: 10.1016/j.tplants.2011.11.002 22209038

[ref3] Ali B. Tao Q. Zhou Y. Gill R. A. Ali S. Rafiq M. T.. (2013). 5-Aminolevolinic acid mitigates the cadmium-induced changes in Brassica napus as revealed by the biochemical and ultra-structural evaluation of roots. Ecotoxicol. Environ. Saf. 92, 271–280. doi: 10.1016/j.ecoenv.2013.02.006, PMID: 23490193

[ref4] Amanullah D. Saud S. Fahad S. (2017). Effects of nitrogen supply on water stress and recovery mechanisms in Kentucky bluegrass. Front. Plant Sci. 8:983. doi: 10.3389/fpls.2017.00983, PMID: 28642781 PMC5463276

[ref5] Antosiewicz D. M. Hennig J. (2004). Overexpression of LCT1 in tobacco enhances the protective action of calcium against cadmium toxicity. Environ. Pollut. 129, 237–245. doi: 10.1016/j.envpol.2003.10.025, PMID: 14987809

[ref6] Arnao M. B. Hernández-Ruiz J. (2019). Melatonin: a new plant hormone and/or a plant master regulator? Trends Plant Sci. 24, 38–48. doi: 10.1016/j.tplants.2018.10.010 30446305

[ref7] Arnon D. I. (1949). Copper enzymes in isolated chloroplasts. Polyphenoloxidase in Beta vulgaris. Plant Physiol. 24, 1–15. doi: 10.1104/pp.24.1.1, PMID: 16654194 PMC437905

[ref8] Axelsen K. B. Palmgren M. G. (2001). Inventory of the superfamily of P-type ion pumps in Arabidopsis. Plant Physiol. 126, 696–706. doi: 10.1104/pp.126.2.696, PMID: 11402198 PMC111160

[ref9] Baldantoni D. Morra L. Zaccardelli M. Alfani A. (2016). Cadmium accumulation in leaves of leafy vegetables. Ecotoxicol. Environ. Saf. 123, 89–94. doi: 10.1016/j.ecoenv.2015.05.019, PMID: 26004982

[ref10] Clemens S. (2006). Toxic metal accumulation, responses to exposure and mechanisms of tolerance in plants. Biochimie 88, 1707–1719. doi: 10.1016/j.biochi.2006.07.003, PMID: 16914250

[ref11] Clemens S. Ma J. F. (2016). Toxic heavy metal and metalloid accumulation in crop plants and foods. Annu. Rev. Plant Biol. 67, 489–512. doi: 10.1146/annurev-arplant-043015-112301, PMID: 27128467

[ref12] Dahab T. El-Aziz N. (2006). Physiological effect of diphenylamin and tryptophan on the growth and chemical constituents of philodendron erubescens plants. World J. Agric. Sci. 2, 75–81.

[ref13] Durenne B. Blondel A. Druart P. Fauconnier M.L. (2017). “Could indolic glucosinolates root profiling be correlated to *Brassica napus* L. cadmium stress tolerance?” in *4th international glucosinolate conference 2017*.

[ref14] Ekmekçi Y. Tanyolaç D. Ayhan B. (2008). Effects of cadmium on antioxidant enzyme and photosynthetic activities in leaves of two maize cultivars. J. Plant Physiol. 165, 600–611. doi: 10.1016/j.jplph.2007.01.017, PMID: 17728009

[ref15] Ellman G. L. (1959). Tissue sulfhydryl groups. Arch. Biochem. Biophys. 82, 70–77. doi: 10.1016/0003-9861(59)90090-6 13650640

[ref16] El-Sayed S. M. Mazhar A. A. M. El-Aziz N. G. A. Mahgoub M. H. Darwish M. A. Shanan N. (2018). Response of *Khaya senegalensis* plants to growth improvement by L-Tryptophan under cadmium stress condition. Middle East J. Agric. 7, 847–857.

[ref17] El-Sayed S. M. Mazhar A. A. M. Mahgoub O. H. El-Aziz N. G. A. Darwish M. A. Shanan N. (2019). Investigation the effect of L-tryptophan on growth and chemical composition of *Eucalyptus gomphocephala* plants under cadmium stress. Middle East J. Agric. 8, 106–116.

[ref18] El-Shanhorey N. A. Ahmed S. S. (2021). Effect of foliar applied tryptophan on tuberose plants for decreasing the harmful effect of some heavy metals pollution in the irrigation water (B) effect of Lead treatments. Alex. J. Agric. Sci. 65, 189–200.

[ref19] Ernst W. Krauss G. J. Verkleij J. Wesenberg D. (2010). Interaction of heavy metals with the Sulphur metabolism in angiosperms from an ecological point of view. Plant Cell Environ. 31, 123–143. doi: 10.1111/j.1365-3040.2007.01746.x 17999660

[ref20] Fahad S. Hussain S. Saud S. Hassan S. Tanveer M. Ihsan M. Z.. (2016). A combined application of biochar and phosphorus alleviates heat-induced adversities on physiological, agronomical and quality attributes of rice. Plant Physiol. Biochem. 103, 191–198. doi: 10.1016/j.plaphy.2016.03.001, PMID: 26995314

[ref21] Fan S. K. Fang X. Z. Guan M. Y. Ye Y. Q. Lin X. Y. Du S. T.. (2014). Exogenous abscisic acid application decreases cadmium accumulation in Arabidopsis plants, which is associated with the inhibition of IRT1-mediated cadmium uptake. Front. Plant Sci. 5:721. doi: 10.3389/fpls.2014.00721, PMID: 25566293 PMC4267193

[ref22] Farooq H. Asghar H. N. Khan M. Y. Saleem M. Zahir Z. A. (2015). Auxin-mediated growth of rice in cadmium-contaminated soil. Turk. J. Agric. For. 39, 272–276. doi: 10.3906/tar-1405-54

[ref23] Gaillard S. Jacquet H. Vavasseur A. Leonhardt N. Forestier C. (2008). AtMRP6/AtABCC6, an ATP-binding cassette transporter gene expressed during early steps of seedling development and up-regulated by cadmium in Arabidopsis thaliana. BMC Plant Biol. 8, 22. doi: 10.1186/1471-2229-8-22, PMID: 18307782 PMC2291051

[ref24] Gu Q. Wang C. A.-O. Xiao Q. Chen Z. A.-O. Han Y. A.-O. (2021). Melatonin confers plant cadmium tolerance: an update. Int. J. Mol. Sci. 22, 11704. doi: 10.3390/ijms222111704, PMID: 34769134 PMC8583868

[ref25] Guo G. Wang Y. Zhang D. Lei M. (2021). Source-specific ecological and health risks of potentially toxic elements in agricultural soils in southern Yunnan Province and associated uncertainty analysis. J. Hazard. Mater. 417:126144. doi: 10.1016/j.jhazmat.2021.126144, PMID: 34229399

[ref26] Halkier B. A. Gershenzon J. (2006). Biology and biochemistry of glucosinolates. Annu. Rev. Plant Biol. 57, 303–333. doi: 10.1146/annurev.arplant.57.032905.105228 16669764

[ref27] Hasan M. K. Ahammed G. J. Sun S. Li M. Yin H. Zhou J. (2019). Melatonin inhibits cadmium translocation and enhances plant tolerance by regulating sulfur uptake and assimilation in *Solanum lycopersicum* L. J. Agric. Food Chem. 67, 10563–10576. doi: 10.1021/acs.jafc.9b02404, PMID: 31487171

[ref28] Hirschi K. D. Korenkov V. D. Wilganowski N. L. Wagner G. J. (2000). Expression of arabidopsis CAX2 in tobacco. Altered metal accumulation and increased manganese tolerance. Plant Physiol. 124, 125–134. doi: 10.1104/pp.124.1.125, PMID: 10982428 PMC59128

[ref29] Hsiao P. Sanjaya Fau-Su R.-C. Fau-Teixeira S. R. da Silva J. A. da Silva T. Ja Fau-Chan M.-T.. (2007). Plant native tryptophan synthase beta 1 gene is a non-antibiotic selection marker for plant transformation. Planta 225, 897–906. doi: 10.1007/s00425-006-0405-y, PMID: 17039373

[ref30] Huybrechts M. Cuypers A. (2019). Cadmium and plant development: an agony from seed to seed. Int. J. Mol. Sci. 20, 3971. doi: 10.3390/ijms20163971, PMID: 31443183 PMC6718997

[ref31] Ismael M. A. Elyamine A. M. Moussa M. G. Cai M. Zhao X. Hu C. (2019). Cadmium in plants: uptake, toxicity, and its interactions with selenium fertilizers. Metallomics 11, 255–277. doi: 10.1039/c8mt00247a, PMID: 30632600

[ref32] Jakovljević T. Fau-Sedak C. M. Sedak M. Fau-Đokić M. Đokić M. Fau-Bilandžić N.. (2013). Balance of glucosinolates content under cd stress in two brassica species. Plant Physiol. Biochem. 63, 99–106. doi: 10.1016/j.plaphy.2012.10.019, PMID: 23254283

[ref33] Jozefczak M. Remans T. Vangronsveld J. Cuypers A. (2012). Glutathione is a key player in metal-induced oxidative stress defenses. Int. J. Mol. Sci. 13, 3145–3175. doi: 10.3390/ijms13033145, PMID: 22489146 PMC3317707

[ref34] Korenkov V. Hirschi K. Crutchfield J. D. Wagner G. J. (2007). Enhancing tonoplast Cd/H antiport activity increases Cd, Zn, and Mn tolerance, and impacts root/shoot Cd partitioning in Nicotiana tabacum L. Planta 226, 1379–1387. doi: 10.1007/s00425-007-0577-0, PMID: 17636324

[ref35] Kumar D. Yusuf M. A. Singh P. Sardar M. Sarin N. B. (2014). Histochemical detection of superoxide and H2O2 accumulation in *Brassica juncea* seedlings. Bio-protocol 4, e 1108. doi: 10.21769/BioProtoc.1108

[ref36] Lasat M. M. Pence N. S. Garvin D. F. Ebbs S. D. Kochian L. V. (2000). The molecular physiology of zinc transport in the Zn hyperaccumulator *Thlaspi caerulescens*. J. Exp. Bot. 51, 71–79. doi: 10.1073/pnas.97.9.4956, PMID: 10938797

[ref37] Lebrazi S. Niehaus K. Bednarz H. Fadil M. Chraibi M. Fikri-Benbrahim K. (2020). Screening and optimization of indole-3-acetic acid production and phosphate solubilization by rhizobacterial strains isolated from Acacia cyanophylla root nodules and their effects on its plant growth. J. Genet. Eng. Biotechnol. 18, 71. doi: 10.1186/s43141-020-00090-2, PMID: 33175273 PMC7658270

[ref38] Li R. Jiang J. Jia S. Zhu X. Su H. Li J. (2019). Overexpressing broccoli tryptophan biosynthetic genes BoTSB1 and BoTSB2 promotes biosynthesis of IAA and indole glucosinolates. Physiol. Plant. 168, 174–187. doi: 10.1111/ppl.12933, PMID: 30706476

[ref39] Liedschulte V. Laparra H. Battey J. N. Schwaar J. D. Broye H. Mark R.. (2017). Impairing both HMA4 homeologs is required for cadmium reduction in tobacco. Plant Cell Environ. 40, 364–377. doi: 10.1111/pce.12870, PMID: 27880006

[ref01] Ludwig-Müller J. Rattunde R. Rößler S. Liedel K. Benade E. Rost A.. (2021). Two auxinic herbicides affect brassica napus plant hormone levels and induce molecular changes in transcription. Biomolecules 11:1153. doi: 10.3390/biom11081153, PMID: 34439819 PMC8391463

[ref40] Mao R. Li W. He Z. Bai Z. Xia P. Liang Z.. (2019). Physiological, transcriptional, and metabolic alterations in spaceflight-subjected *Senna obtusifolia*. Plant Physiol. Biochem. 139, 33–43. doi: 10.1016/j.plaphy.2019.03.009, PMID: 30878836

[ref41] Mills R. F. Francini A. Ferreira da Rocha P. S. Baccarini P. J. Aylett M. Krijger G. C.. (2005). The plant P1B-type ATPase AtHMA4 transports Zn and Cd and plays a role in detoxification of transition metals supplied at elevated levels. FEBS Lett. 579, 783–791. doi: 10.1016/j.febslet.2004.12.040, PMID: 15670847

[ref42] Morel M. Crouzet J. Gravot A. Auroy P. Leonhardt N. Vavasseur A.. (2009). AtHMA3, a P1B-ATPase allowing Cd/Zn/Co/Pb vacuolar storage in Arabidopsis. Plant Physiol. 149, 894–904. doi: 10.1104/pp.108.130294, PMID: 19036834 PMC2633814

[ref43] Nounjan N. Nghia P. T. Theerakulpisut P. (2012). Exogenous proline and trehalose promote recovery of rice seedlings from salt-stress and differentially modulate antioxidant enzymes and expression of related genes. J. Plant Physiol. 169, 596–604. doi: 10.1016/j.jplph.2012.01.004, PMID: 22317787

[ref44] Pang Q. Chen S. Yan X. (2009). Characterization of glucosinolate--myrosinase system in developing salt cress *Thellungiella halophila*. Physiol. Plant. 136, 1–9. doi: 10.1111/j.1399-3054.2009.01211.x, PMID: 19508363

[ref45] Paunov M. Koleva L. Vassilev A. Vangronsveld J. (2018). Effects of different metals on photosynthesis: cadmium and zinc affect chlorophyll fluorescence in durum wheat. Int. J. Mol. Sci. 19, 787. doi: 10.3390/ijms19030787, PMID: 29522461 PMC5877648

[ref46] Rehman M. Z. Rizwan M. Ghafoor A. Naeem A. Ali S. Sabir M.. (2015). Effect of inorganic amendments for in situ stabilization of cadmium in contaminated soils and its phyto-availability to wheat and rice under rotation. Environ. Sci. Pollut. Res. Int. 22, 16897–16906. doi: 10.1007/s11356-015-4883-y, PMID: 26109220

[ref47] Rizwan M. Ali S. Zia Ur Rehman M. Rinklebe J. Tsang D. C. W. Bashir A.. (2018). Cadmium phytoremediation potential of brassica crop species: a review. Sci. Total Environ. 631–632, 1175–1191. doi: 10.1016/j.scitotenv.2018.03.104, PMID: 29727943

[ref48] Rizwan M. Meunier J. D. Miche H. Keller C. (2012). Effect of silicon on reducing cadmium toxicity in durum wheat (*Triticum turgidum* L. cv. Claudio W.) grown in a soil with aged contamination. J. Hazard. Mater. 209-210, 326–334. doi: 10.1016/j.jhazmat.2012.01.033, PMID: 22301080

[ref49] Romero-Puertas M. C. Corpas F. J. Rodríguez-Serrano M. Gómez M. Del Río L. A. Sandalio L. M. (2007). Differential expression and regulation of antioxidative enzymes by cadmium in pea plants. J. Plant Physiol. 164, 1346–1357. doi: 10.1016/j.jplph.2006.06.018, PMID: 17074418

[ref50] Sandalio L. M. Dalurzo H. C. Gómez M. Romero-Puertas M. C. del Río L. A. (2001). Cadmium-induced changes in the growth and oxidative metabolism of pea plants. J. Exp. Bot. 52, 2115–2126. doi: 10.1093/jexbot/52.364.2115, PMID: 11604450

[ref51] Sardar R. Ahmed S. Shah A. A. Yasin N. A. (2022). Selenium nanoparticles reduced cadmium uptake, regulated nutritional homeostasis and antioxidative system in *Coriandrum sativum* grown in cadmium toxic conditions. Chemosphere 287:132332. doi: 10.1016/j.chemosphere.2021.132332, PMID: 34563771

[ref52] Saud S. Li X. Yang C. Zhang L. Fahad S. Saddam H.. (2014). Silicon application increases drought tolerance of Kentucky bluegrass by improving plant water relations and morphophysiological functions. Sci. World J. 2014, 368694. doi: 10.1155/2014/368694, PMID: 25054178 PMC4098892

[ref53] Saud S. Yajun C. Fahad S. Hussain S. Na L. Xin L.. (2016). Silicate application increases the photosynthesis and its associated metabolic activities in Kentucky bluegrass under drought stress and post-drought recovery. Environ. Sci. Pollut. Res. Int. 23, 17647–17655. doi: 10.1007/s11356-016-6957-x, PMID: 27236444

[ref54] Sun H. Dai H. Wang X. Wang G. (2016). Physiological and proteomic analysis of selenium-mediated tolerance to Cd stress in cucumber (*Cucumis sativus* L.). Ecotoxicol. Environ. Saf. 133, 114–126. doi: 10.1016/j.ecoenv.2016.07.003, PMID: 27434422

[ref55] Takahashi R. Ishimaru Y. Nakanishi H. Nishizawa N. K. (2011). Role of the iron transporter OsNRAMP1 in cadmium uptake and accumulation in rice. Plant Signal. Behav. 6, 1813–1816. doi: 10.4161/psb.6.11.17587, PMID: 22067109 PMC3329356

[ref56] Verret F. Gravot A. Auroy P. Leonhardt N. David P. Nussaume L.. (2004). Overexpression of AtHMA4 enhances root-to-shoot translocation of zinc and cadmium and plant metal tolerance. FEBS Lett. 576, 306–312. doi: 10.1016/j.febslet.2004.09.023, PMID: 15498553

[ref57] Volkov R. A. Panchuk I. I. Mullineaux P. M. Schöffl F. (2006). Heat stress-induced H(2)O (2) is required for effective expression of heat shock genes in Arabidopsis. Plant Mol. Biol. 61, 733–746. doi: 10.1007/s11103-006-0045-4, PMID: 16897488

[ref58] Wang L. Wang Y. Felix G. (2019). Peptide feeding and mechanical wounding for tomato seedlings. Bio-protocol 9:e3194. doi: 10.21769/BioProtoc.3194, PMID: 33654993 PMC7854076

[ref59] Wong C. K. E. Cobbett C. S. (2009). HMA P-type ATPases are the major mechanism for root-to-shoot cd translocation in *Arabidopsis thaliana*. New Phytol. 181, 71–78. doi: 10.1111/j.1469-8137.2008.02638.x, PMID: 19076718

[ref60] Yao M. Ge W. Zhou Q. Zhou X. Luo M. Zhao Y.. (2021). Exogenous glutathione alleviates chilling injury in postharvest bell pepper by modulating the ascorbate-glutathione (AsA-GSH). Cycle 352:129458. doi: 10.1016/j.foodchem.2021.129458, PMID: 33714166

[ref61] Zemanová V. Pavlík M. Pavlíková D. Tlusto P. (2014). The significance of methionine, histidine and tryptophan in plant responses and adaptation to cadmium stress. Plant Soil Environ. 60, 426–432. doi: 10.17221/544/2014-PSE

[ref62] Zhan F. Zeng W. Yuan X. Li B. Li T. Zu Y.. (2019). Field experiment on the effects of sepiolite and biochar on the remediation of cd- and Pb-polluted farmlands around a Pb-Zn mine in Yunnan Province, China. Environ. Sci. Pollut. Res. Int. 26, 7743–7751. doi: 10.1007/s11356-018-04079-w, PMID: 30671759

[ref63] Zhang Z. Huang R. (2013). Analysis of malondialdehyde, chlorophyll Proline, soluble sugar, and glutathione content in Arabidopsis seedling. Bio-protocol 3:e817. doi: 10.21769/BioProtoc.817

[ref64] Zhao F. J. Zhang W. W. Song J. F. Yue S. Q. Duan K. X. Yang H. Q. (2018). MhMAPK4 from Malus hupehensis Rehd. Decreases cell death in tobacco roots by controlling Cd2+ uptake. Ecotoxicol. Environ. Saf. 168, 230–240. doi: 10.1016/j.ecoenv.2018.09.126, PMID: 30388541

