# Supplementary material for: Exogenous tryptophan application improves cadmium tolerance and inhibits cadmium upward transport in broccoli (Brassica oleracea var. italica)
Source: Front Plant Sci. 2022 Aug 11;13:969675. doi: 10.3389/fpls.2022.969675 (PMC9403758; doi:10.3389/fpls.2022.969675)
Supplement: Supplementary file 1 [file Data_Sheet_1.ZIP › Supplementary/Supplementary Table 2.docx]

**Table S2** Primer pairs used for qRT-PCR analysis

| Genes | Forward primers (5´→3´) | Reverse primers (5´→3´) |
| --- | --- | --- |
| *ACTIN* | TGTGACGTGGATATCAGGAAGGAC | GAACCACCGATCCAGACACTGTACT |
| *CYP79B2* | ACATTCGCTTTTTGCATCTCTGACT | CTCTTTCCTTCTCTCCACATCTTGA |
| *YUCCA8* | GGGAGAGAATGCAGAGAGAGTGG | CTTAGCGAAGTGATTAGCGAGGT |
| *NIT2* | GTTTCTCCTCCCTCAACCACCA | GCAAAATCAACAATCCCCCAA |
| *SOT16* | AACCCGCTCCTCAAACGGAAC | CCTTGGGGTCTCTCCAGATGTAAAC |
| *ZIP1* | CGCCTTGTCTTGAGACCACT | ATACGCCGTCGCGAATGTAT |
| *IRT1* | TACATGGCGCTTGTGGATCT | CATGCCACCACACCCCAAAA |
| *NRAMP2* | ACAACCGCTTATGTTGCCTTT | GGCCTGAAGGGGCAGTTATT |
| *XTH3* | TGGGCTTCTTTGGAACCCTT | ACACACTATCGCTATTTGTCGTC |
| *HMA3* | TGGCCCTAAGGACAAGCAAA | TGCATCTTCGGGCCACAAT |
| *CAX2* | TGGGGTCGGTTAATGAACTTG | GCGTGTTCAGGGAAACTCAG |
| *CAX4* | TTGCCTTGTCCATCCTCGTC | TGTGACCTTACTGAATACCCTGA |
| *MRP3* | CACCAAAGTGGCCGTATCCT | GTAAAGCTTGGAGAGCACGC |
| *HMA2* | GTGCTCTTACCAAGGCAGC | TGCTTGAGACCCAGTAAAGTAAA |
| *HMA4* | TAGCTGTTGCGCTGAGAAGA | TCCCCAAGAATCTGCATGTCT |
